# Fish Consumption, Bone Mineral Density, and Risk of Hip Fracture Among Older Adults: The Cardiovascular Health Study

**DOI:** 10.1002/jbmr.87

**Published:** 2010-06-23

**Authors:** Jyrki K Virtanen, Dariush Mozaffarian, Jane A Cauley, Kenneth J Mukamal, John Robbins, David S Siscovick

**Affiliations:** 1 University of Eastern Finland, Kuopio Campus, Institute of Public Health and Clinical Nutrition Kuopio, Finland; 2 Division of Cardiovascular Medicine and Channing Laboratory, Brigham and Women's Hospital and Harvard Medical School, and Departments of Epidemiology and Nutrition, Harvard School of Public Health Boston, MA, USA; 3 Department of Epidemiology, University of Pittsburgh Pittsburgh, PA, USA; 4 Department of Medicine, Beth Israel Deaconess Medical Center Boston, MA, USA; 5 Department of Internal Medicine, University of California at Davis School of Medicine Sacramento, CA, USA; 6 Cardiovascular Health Research Unit, Departments of Medicine and Epidemiology, University of Washington Seattle, WA, USA

**Keywords:** bone mineral density, fatty acids, fish, hip fracture, population studies

## Abstract

Marine *n*-3 polyunsaturated fatty acids (PUFAs) eicosapentaenoic acid (EPA) and docosahexaenoic acid (DHA) may be beneficial for bone health, but few studies have investigated the association with fish consumption. Our aim was to study associations of fish and EPA + DHA consumption with bone mineral density (BMD) and hip fracture risk and determine whether high linoleic acid (LA) intake, the major dietary *n*-6 PUFA, modifies the associations. The study population consisted of 5045 participants aged 65 years and older from the Cardiovascular Health Study. Data on BMD were available for 1305 participants. Food-frequency questionnaire was used to assess dietary intake, and hip fracture incidence was assessed prospectively by review of hospitalization records. After multivariable adjustment, femoral neck BMD was 0.01 g/cm^2^ lower in the highest versus lowest tuna/other-fish intake category (*p* = .05 for trend). EPA + DHA intake (higher versus lower median of 0.32 g/day) was associated with lower femoral neck BMD (0.66 versus 0.71 g/cm^2^, *p* < .001) among those with LA intake greater than the median 12.1 g/day (*p* = .03 for interaction). No significant associations were found with total-hip BMD. During mean follow-up of 11.1 years, 505 hip fractures occurred. Fish or EPA + DHA consumption was not significantly associated with fracture incidence [hazard ratio (HR) for extreme categories: HR = 1.23, 95% confidence interval (CI) 0.83–1.84 for tuna/other fish; HR = 1.16, 95% CI 0.91–1.49 for fried fish; and HR = 0.98, 95% CI 0.71–1.36 for EPA + DHA]. High LA intake did not modify these associations. In this large prospective cohort of older adults, fish consumption was associated with very small differences in BMD and had no association with hip fracture risk. © 2010 American Society for Bone and Mineral Research.

## Introduction

Polyunsaturated fatty acids (PUFAs) have an important role in the prevention of chronic diseases. The two classes of PUFAs, *n*-3 and *n*-6 fatty acids, are not interconvertible in mammalian cells and are both indispensable for humans. Considerable evidence supports benefits of absolute intakes of *n*-3 PUFAs(1) and also likely *n*-6 PUFAs(2) for cardiovascular disease (CVD). Imbalance between relative intakes of *n*-3 and *n*-6 PUFAs has been suggested to increase the risk of chronic diseases such as CVD and cancer,(3) but evidence from human studies is scarce.(4)

*n*-3 and *n*-6 PUFAs also may affect bone health. In animal models, diets high in eicosapentaenoic acid (EPA) and docosahexaenoic acid (DHA), the long-chain *n*-3 PUFA derived from seafood, have been shown to attenuate bone loss in ovariectomized animals compared with diets enriched in *n*-6 PUFAs.(5) Although the exact mechanisms are not known, *n*-6 and *n*-3 PUFAs have been proposed to have different effects on factors affecting bone formation and resorption, such as prostaglandins, calcium, and cytokines.(5,6) However, few studies have been published about the effects of fish or EPA + DHA consumption on bone health in humans. Small intervention trials in postmenopausal women with fish oil supplementation have yielded mixed results.(7–9) In recent cross-sectional studies, high intakes of seafood or *n*-3 PUFAs have been associated with greater bone mineral density (BMD) in some(10–12) but not all study populations,(13) whereas a higher dietary *n*-6:*n*-3 PUFA ratio has been associated with lower BMD.(14) In a Japanese case-control study, moderate fish consumption was associated with lower hip fracture risk.(15) In prospective studies, serum long-chain *n*-3 PUFAs, especially DHA, have been positively associated with bone mineral accrual and peak BMD in young men.(16) Consumption of dark-meat (oily) fish was associated with a lower incidence of hip fractures in the Nurses' Health Study,(17) but no association with a risk of bone fracture was found in fish eaters compared with meat eaters in the EPIC-Oxford Study(18) or in a Japanese cohort.(19)

Thus relatively few studies have investigated the relationship of fish or *n*-3 PUFA consumption with bone health in humans, and that ones that have show conflicting results. Furthermore, risk of hip fracture is by far the most perilous consequence of osteoporosis in older adults, but few studies have focused on this population. The purpose of this study was to investigate the association of fish and estimated EPA + DHA consumption with BMD and incidence of hip fractures among older men and women in the Cardiovascular Health Study, a National Heart, Lung, and Blood Institute (NHLBI)–sponsored prospective cohort study of older adults. Because *n*-6 and *n*-3 PUFAs may have different effects on bone,(5,6) we also investigated whether linoleic acid (LA) intake, the major *n*-6 PUFA in the diet, modified the associations with fish or EPA + DHA intake.

## Materials and Methods

### Design and population

The design and recruitment experience of the Cardiovascular Health Study have been described previously.(20,21) Briefly, 5201 men and women aged 65 years or older (mean 72.8 years, min-max 65 to 100 years) at baseline were randomly selected and enrolled in 1989–1990 from Medicare eligibility lists in four US communities. An additional 687 black participants who were enrolled in 1992–1993 were not included in this analysis owing to lack of dietary assessment in this group at baseline. Each center's institutional review committee approved the study, and all subjects gave informed consent. All participants underwent extensive baseline evaluations, including standard questionnaires, physical examination, performance measures, and laboratory testing.(20,21) Parts of the baseline evaluation were repeated during annual follow-up visits. Prevalent coronary heart disease, stroke, transient ischemic attack, hypertension, and diabetes were defined using patients' reports and confirmed by centralized review of hospital and clinic records.(20,21)

### Dietary assessment

Usual dietary intakes were assessed in 1989–1990 using a picture-sort version of the National Cancer Institute food-frequency questionnaire (FFQ).(22) Participants were asked to indicate how often, on average, they had consumed various foods during the past year, including tuna fish, other broiled or baked fish, and fried fish or fish sandwiches (“fish burgers”). Nutrient intakes were estimated from questionnaire responses and adjusted for total calories using regression analyses(23,24); a semiquantitative picture-sort version was validated against 24-hour dietary recalls.(22) Dietary EPA and DHA intakes were calculated from questionnaire responses using estimated fish and shellfish serving sizes [3 to 5 oz (84 to 140 g)](25) and US commercial landings data.(26) Tuna or other broiled or baked fish correlated with combined plasma phospholipid EPA + DHA concentrations (*r* = 0.51), a biomarker of *n*-3 PUFA intake, in a subsample of participants.(27) Phospholipid EPA + DHA concentrations did not correlate with fried fish consumption (*r* = 0.04), consistent with observation that lean types of fish typically are fried (eg, cod and pollock).(27) Consistent with prior reports of CVD risk in this study population,(27,28) we separately evaluated consumption of tuna/other fish versus fried fish.

### Measurement of bone mineral density

In 1994–1995, 1591 participants at the Sacramento and Pittsburgh clinic sites underwent routine dual-energy X-ray absorptiometry (DXA) scans; complete data were available subsequently for 1567 subjects. Compared with those who were not screened, the screened group had less white participants; were healthier and more physically active; had higher cognitive score, education, and income; and used more alcohol, calcium, multivitamins, estrogen, and thyroid medications and fewer benzodiazepine and thiazide medications.(29) BMD was measured with QDR-2000 bone densitometers (DXA; Hologic, Bedford, MA, USA) according to a written protocol. Scans were performed locally, with independent external quality assurance, and read blindly at the University of California, San Francisco, using Hologic software, as described previously.(30) We used BMD (in g/cm^2^) of the total hip and femoral neck as our primary measures of BMD.

### Determination of hip fracture

Details of the Cardiovascular Health Study (CHS) protocol for identification of hip fracture have been published previously.(29) The CHS investigators conducted active and passive surveillance to capture all hospitalizations for each participant. Participants reported hospitalizations and other acute events at annual clinic visits and interim telephone interviews, and discharge summaries and diagnoses were obtained for all hospitalizations. These records were supplemented with information from the Centers for Medicare and Medicaid Services (CMS) health care utilization database for hospitalizations. We defined hip fracture by a hospital discharge *International Classification of Diseases*, Ninth Revision (ICD-9), code of 820.xx without a concomitant code for motor vehicle accident (E810–E819) or pathologic fracture (733.1x). Hip fractures that occurred from the study entry to June 30, 2003, were included. The average follow-up time was 11.1 years (range 0.1 to 17.6 years).

### Other risk factors

At baseline, participants were asked whether they had frequent falls in the past year; no specific prompt regarding the definition of *frequent falls* was given. Participants also self-reported whether they had arthritis and whether they had difficulty arising from a bed or chair. We defined hypertension, orthostatic hypotension, and diabetes with standard criteria, as described previously.(31,32) Field center staff directly measured weight, standing height, and waist circumference. Leisure-time physical activity was assessed as a weighted sum of kilocalories expended in specific physical activities.(32) Clinical CVD included confirmed coronary heart disease, congestive heart failure, cerebrovascular disease, and peripheral vascular disease.(33,34) Psychoactive medication use included antidepressants, benzodiazepines, and antipsychotic agents. Depressive symptoms were assessed at baseline with the CES-D scale.(35)

### Statistical analysis

After excluding individuals with missing information on fish or EPA + DHA consumption, 1305 participants were available for analyses of BMD and 5045 participants for analyses of incident hip fracture. The cross-sectional relationship of fish consumption and EPA + DHA or LA intake with BMD was assessed with generalized linear models and with incident hip fracture using Cox proportional hazards, with follow-up from 1989 until 2003 and censoring at the first event of hip fracture, death, or the latest date of follow-up until June 30, 2003. The initial models in all analyses were adjusted for age, sex, race (white versus other), weight, and height. The multivariable-adjusted models further included smoking, physical activity, education, and intakes of alcohol (drinks/week), protein (percent of energy), and fruits (servings/day). Further adjustments for clinic, frequent falls, self-reported difficulty getting out of bed or chair, visual problems, arthritis, diabetes, cancer, CVD, hypertension, depression, systolic and diastolic blood pressure, weight change of more than10 lb during previous year, weight at age 50, use of estrogen (in women only), thiazide diuretics, thyroid agents, corticosteroids or psychoactive medication, and intakes of energy, beef or pork, dairy, vegetables, total or saturated fat, calcium, phosphate, or fish oil supplements (<5% of participants) did not change the associations (<5% change in the hazard ratio). Tests of linear trend were conducted by assigning the median values for each category of exposure variable and treating those as a single continuous variable. Linear (for continuous variables) or logistic (for binary variables) regression was used to evaluate the trend. Stratified analyses and likelihood-ratio tests using multiplicative interaction terms were used to explore potential effect modification by gender and LA intake and in post hoc analyses by age, physical activity, and calcium intake. All *p* values were two-tailed (α = 0.05). Data were analyzed using SPSS 14.0 for Windows (SPSS, Inc., Chicago, IL, USA).

## Results

### Participant characteristics at baseline

At baseline, the mean (±SD) intake of tuna/other fish was 1.6 ± 1.5 servings/week, fried fish 0.5 ± 0.7 servings/week, and EPA + DHA 0.29 ± 0.24 g/day. Higher tuna/other-fish consumption was associated with younger age, female sex, lower smoking rates, higher education, lower use of psychoactive medication, and higher use of thyroid agents, whereas higher fried-fish consumption was associated with male sex, nonwhite race, higher BMI, lower education, higher prevalence of CVD and diabetes, difficulties in arising from bed or chair, and lower use of estrogen (in women) and thyroid agents (Table 1). Tuna/other-fish consumption also was associated with higher intakes of energy, alcohol, dairy, fruits, vegetables, protein, calcium, and EPA + DHA and lower intakes of LA. Fried-fish consumption was positively associated with intakes of energy, dairy, beef or pork, vegetables, protein, calcium, total and saturated fat, LA, and EPA + DHA and inversely associated with alcohol intake.

**Table 1:** Participant Characteristics According to Fish Consumption

		Tuna/other fish (servings)				Fried fish (servings)^a^		
			
	Whole population (*n* = 5045)	<1/month (*n* = 535)	1–3/month (*n* = 1189)	1–2/week (*n* = 2352)	≥3/week (*n* = 969)	<1/month (*n* = 2422)	1–3/month (*n* = 1630)	≥1/week (*n* = 993)
Age (years)	72.8 ± 5.6	74.2 ± 6.6	73.3 ± 5.7	72.6 ± 5.4	71.8 ± 5.1^b^	72.9 ± 5.6	72.5 ± 5.3	73.1 ± 5.7
Men (%)	43	47	47	43	35^b^	38	46	51^b^
White race (%)	95	94	95	95	96	96	95	91^b^
Current smoker (%)	12	15	14	11	10^b^	12	11	12
Education ≥ high school diploma (%)	72	51	67	76	82^b^	79	69	62^b^
Leisure-time physical activity (kcal/week)	1230 ± 1630	1060 ± 1670	1190 ± 1590	1280 ± 1690	1230 ± 1510	1200 ± 1570	1220 ± 1600	1290 ± 1820
Body mass index (kg/m^2^)	26.4 ± 4.5	26.0 ± 4.7	26.3 ± 4.6	26.4 ± 4.4	26.7 ± 4.6	26.1 ± 4.5	26.7 ± 4.4	26.8 ± 4.7^b^
Dietary intake								
Energy (kcal/day)	1820 ± 660	1630 ± 660	1620 ± 600	1860 ± 640	2100 ± 680^b^	1620 ± 560	1850 ± 610	2260 ± 750^b^
Dairy (servings/day)	1.1 ± 0.6	1.0 ± 0.6	1.0 ± 0.5	1.1 ± 0.6	1.2 ± 0.6^b^	1.0 ± 0.6	1.1 ± 0.6	1.2 ± 0.6^b^
Beef or pork (servings/day)	0.9 ± 0.7	0.9 ± 0.8	0.8 ± 0.7	0.9 ± 0.7	0.8 ± 0.7	0.7 ± 0.6	0.9 ± 0.6	1.2 ± 0.8^b^
Fruits (servings/day)	2.1 ± 1.1	1.8 ± 1.0	1.8 ± 1.0	2.2 ± 1.1	2.5 ± 1.1^b^	2.2 ± 1.1	2.1 ± 1.1	2.2 ± 1.1
Vegetables (servings/day)	2.5 ± 1.4	1.9 ± 1.3	1.9 ± 1.2	2.5 ± 1.2	3.3 ± 1.5^b^	2.4 ± 1.3	2.4 ± 1.3	2.7 ± 1.5^b^
Alcohol (drinks/week)	2.6 ± 6.2	1.6 ± 5.4	2.4 ± 6.1	2.8 ± 6.6	2.7 ± 5.9^b^	2.7 ± 6.1	2.5 ± 6.5	2.3 ± 6.0^b^
Protein (% of energy)	17.6 ± 2.9	16.1 ± 3.1	16.4 ± 2.7	17.8 ± 2.7	19.4 ± 2.5^b^	17.2 ± 3.1	17.4 ± 2.6	19.0 ± 2.5^b^
Calcium (mg/day)	860 ± 358	734 ± 326	766 ± 332	885 ± 351	989 ± 371^b^	801 ± 339	878 ± 350	974 ± 387^b^
EPA + DHA (g/day)	0.29 ± 0.24	0.05 ± 0.04	0.12 ± 0.06	0.30 ± 0.15	0.59 ± 0.29^b^	0.28 ± 0.26	0.25 ± 0.19	0.37 ± 0.24^b^
Linoleic acid (% of energy)	6.5 ± 2.2	6.6 ± 2.4	6.6 ± 2.3	6.4 ± 2.2	6.5 ± 1.9^b^	6.2 ± 2.4	6.6 ± 2.0	7.1 ± 1.9^b^
History of (%)								
Cardiovascular disease	25	27	26	25	25	24	25	29^b^
Diabetes	15	16	17	15	14	14	15	18^b^
Hypertension	42	44	43	42	41	41	43	44
Cancer	15	13	16	15	16	15	15	15
Arthritis	51	50	53	52	49	51	53	52
Frequent falls	3	5	3	3	3	3	3	3
Difficulty arising from bed or chair	4	5	6	4	4	4	4	6^b^
Use of (%)								
Psychoactive medication	16	21	16	15	16^b^	16	15	17
Estrogen (women)	12	10	11	12	15	15	10	10^b^
Thyroid agents	9	5	8	10	11^b^	10	8	7^b^
Thiazide diuretics	19	20	19	19	18	18	18	21

Values are means ± SD (continuous variables) or frequencies (categorical variables).

a The two highest categories of fried-fish consumption were combined because only 68 subjects consumed fried fish 3 or more times per week.

b *p* < .05 for trend across categories of intake, adjusted for age.

### Consumption of fish or EPA + DHA and bone mineral density

Table 2 shows the average BMD of the total hip and femoral neck according to the baseline consumption of fish or EPA + DHA, after multivariable adjustments for variables that changed the associations. In general, the differences between the groups were modest. The only statistically significant association was observed between a more frequent tuna/other-fish consumption and a slightly lower average BMD at the femoral neck (0.01 g/cm^2^ difference between the highest and lowest quintiles, *p* = .05 for trend). However, despite a statistically significant linear trend, a dose-response relationship was not apparent: The highest BMD was observed in those consuming 1 to 3 servings/month and 1 to 2 servings/week (Table 2). Gender did not modify the associations (Table 2). Stratified by the median LA intake of 12.1 g/day, higher EPA + DHA intake (≥median 0.32 versus <0.32 g/day) was associated with a slightly lower average femoral neck BMD (0.66 versus 0.71 g/cm^2^, *p* < .001 for difference) and total-hip BMD (0.79 versus 0.83 g/cm^2^, *p* < .001) among those with LA intake above the median. No differences in either femoral neck BMD (0.68 versus 0.69 g/cm^2^, *p* = .77, *p* = .03 for interaction) or total-hip BMD (0.81 versus 0.81 g/cm^2^, *p* = .67, *p* = .08 for interaction) were found between higher and lower EPA + DHA intake when the LA intake was below the median. LA intake did not modify the associations between tuna/other-fish or fried-fish consumption (stratified as ≥ versus <1 time/week) and either femoral neck or total-hip BMD (*p* > .10 for interactions).

**Table 2:** Multivariable-Adjusted Bone Mineral Densities According to Fish and EPA + DHA Consumption

	Tuna/other fish (servings)					
			
	<1/month (*n* = 63)	1–3/month (*n* = 213)	1–2/week (*n* = 637)	≥3/week (*n* = 391)	*p* for trend	*p* for interaction
Total hip, all	0.81 (0.77, 0.84)	0.81 (0.79, 0.83)	0.82 (0.81, 0.83)	0.80 (0.79, 0.82)	.38	
Men (*n* = 560)	0.93 (0.87, 0.98)	0.94 (0.90, 0.97)	0.93 (0.91, 0.94)	0.93 (0.90, 0.95)	.81	.09
Women (*n* = 744)	0.72 (0.68, 0.77)	0.72 (0.70, 0.75)	0.74 (0.73, 0.75)	0.71 (0.69, 0.73)	.24	
Femoral neck, all	0.68 (0.65, 0.71)	0.70 (0.68, 0.72)	0.69 (0.68, 0.70)	0.67 (0.66, 0.68)	.05	
Men (*n* = 560)	0.77 (0.72, 0.82)	0.79 (0.76, 0.82)	0.76 (0.75, 0.78)	0.76 (0.73, 0.78)	.21	.15
Women (*n* = 744)	0.62 (0.58, 0.66)	0.63 (0.61, 0.65)	0.64 (0.62, 0.65)	0.61 (0.59, 0.62)	.09	

	Fried fish (servings)^a^				
			
	<1/month (*n* = 713)	1–3/month (*n* = 374)	≥1/week (*n* = 217)	*p* for trend	*p* for interaction
Total hip, all	0.81 (0.80, 0.82)	0.82 (0.81, 0.84)	0.80 (0.78, 0.82)	.63	
Men (*n* = 560)	0.93 (0.91, 0.95)	0.94 (0.92, 0.96)	0.91 (0.88, 0.94)	.54	.69
Women (*n* = 744)	0.73 (0.71, 0.74)	0.73 (0.72, 0.75)	0.71 (0.69, 0.74)	.73	
Femoral neck, all	0.68 (0.67, 0.69)	0.69 (0.68, 0.71)	0.68 (0.67, 0.70)	0.74	
Men (*n* = 560)	0.76 (0.75, 0.78)	0.78 (0.76, 0.80)	0.76 (0.74, 0.79)	0.75	.30
Women (*n* = 744)	0.63 (0.61, 0.64)	0.63 (0.61, 0.65)	0.62 (0.60, 0.64)	0.81	

	EPA + DHA (mg/day)						
			
	<145 (*n* = 261)	145–229 (*n* = 261)	230–411 (*n* = 260)	412–519 (*n* = 261)	>519 (*n* = 261)	*p* for trend	*p* for interaction
Total hip, all	0.81 (0.79, 0.83)	0.83 (0.81, 0.84)	0.82 (0.80, 0.84)	0.81 (0.79, 0.82)	0.80 (0.79, 0.82)	.27	
Men (*n* = 560)	0.94 (0.91, 0.97)	0.92 (0.89, 0.95)	0.94 (0.91, 0.96)	0.92 (0.89, 0.95)	0.92 (0.89, 0.95)	.36	.50
Women (*n* = 744)	0.72 (0.70, 0.74)	0.74 (0.72, 0.77)	0.73 (0.71, 0.75)	0.72 (0.70, 0.74)	0.72 (0.70, 0.74)	.42	
Femoral neck, all	0.69 (0.67, 0.70)	0.70 (0.68, 0.71)	0.70 (0.68, 0.71)	0.68 (0.66, 0.69)	0.68 (0.66, 0.69)	.12	
Men (*n* = 560)	0.78 (0.75, 0.80)	0.76 (0.73, 0.78)	0.78 (0.76, 0.81)	0.76 (0.74, 0.79)	0.75 (0.73, 0.78)	.37	.23
Women (*n* = 744)	0.63 (0.61, 0.64)	0.64 (0.62, 0.66)	0.63 (0.61, 0.65)	0.62 (0.60, 0.63)	0.61 (0.60, 0.63)	.15	

### Consumption of fish or EPA + DHA and risk of hip fracture

During 11.1 years of follow-up, 371 women (12.9%) and 134 men (6.2%) experienced a hip fracture. In the overall population, consumption of tuna/other fish, fried fish, or EPA + DHA was not associated with risk of hip fracture (Table 3). No significant interactions were evident by gender (*p* ≥ .20 for interactions). We also assessed the relationship between fish or EPA + DHA consumption and fracture risk among those with a possibly higher risk of fractures, that is, those with a higher age or low physical activity or low intake of calcium (all stratified by median). However, no effect modification was found in any case (*p* > .10 for interactions). We were not able to assess the interaction with race because only 26 African-American participants experienced a hip fracture. We did not find evidence that high LA intake would modify the associations between fish or EPA + DHA consumption and risk of hip fracture (*p* > .10 for interactions). Dietary habits may change over time, so use of a single FFQ at baseline may cause misclassification in the fish-consumption estimates because of the long follow-up. However, the associations between fish or EPA + DHA consumption and hip fracture risk were not appreciably different in those with 5 years or less of follow-up compared with those with more than 5 years of follow-up (data not shown).

**Table 3:** Risk of Hip Fracture According to Fish and EPA + DHA Consumption

	Tuna/other fish (servings)				
		
	<1/month (*n* = 535)	1–3/month (*n* = 1189)	1–2/week (*n* = 2352)	≥3/week (*n* = 969)	*p* for trend
No. of cases (%)	45 (8.4)	121 (10.2)	248 (10.5)	91 (9.4)	
Model 1	1	1.15 (0.82–1.62)	1.14 (0.83*–*1.5)	1.01 (0.70–1.46)	.85
Model 2	1	1.22 (0.86–1.72)	1.30 (0.93*–*1.82)	1.23 (0.83–1.84)	.32

	Fried fish (servings)^a^			
		
	<1/month (*n* = 2422)	1–3/month (*n* = 1630)	≥1/week (*n* = 993)	*p* for trend
No. of cases (%)	261 (10.8)	147 (9.0)	97 (9.8)	
Model 1	1	0.94 (0.76–1.15)	1.15 (0.90–1.46)	.45
Model 2	1	0.94 (0.76–1.15)	1.16 (0.91–1.49)	.43

	EPA + DHA (mg/day)					
			
	<93 (*n* = 1009)^†^	93–178 (*n* = 1009)^†^	179–275 (*n* = 1009)^†^	275–475 (*n* = 1009)^†^	>475 (*n* = 1009)	*p* for trend
No. of cases (%)	97 (9.6)	109 (10.8)	107 (10.6)	107 (10.6)	85 (8.4)	
Model 1	1	1.23 (0.93–1.62)	1.11 (0.84–1.46)	1.15 (0.87–1.51)	0.87 (0.65–1.18)	.23
Model 2	1	1.31 (0.99–1.73)	1.18 (0.89–1.56)	1.29 (0.96–1.75)	0.98 (0.71–1.36)	.93

Values are hazard ratio (95% CI). Model 1: Adjusted for age, sex, race, height, and weight. Model 2: Adjusted for model 1 and smoking, physical activity, education, and intakes of alcohol, protein, fruits, and tuna/other fish or fried fish (in analyses with fish intake only).

a The two highest categories of fried-fish consumption were combined because only 68 subjects consumed fried fish 3 or more servings/week.

## Discussion

Neither fish consumption nor estimated EPA + DHA consumption was significantly associated with a lower incidence of hip fracture in this prospective, community-based study of older adults, a population with a potentially high risk for osteoporotic fractures. Higher tuna/other-fish intake was associated with slightly lower BMD at the femoral neck, but the difference was very modest, and a dose-response relationship was not apparent.

There are plausible mechanisms whereby high *n*-3 PUFA intake or a low *n*-6:*n*-3 PUFA ratio could affect bone metabolism. For example, the major prostaglandin involved in bone metabolism, prostaglandin E_2_, which is synthesized from an *n*-6 PUFA, arachidonic acid, stimulates bone formation in low concentrations but is inhibitory in high concentrations.(5) *n*-3 PUFAs inhibit production of proinflammatory cytokines, such as interleukin 1 and tumor necrosis factor α,(36) which have been implicated as mediators of postmenopausal bone loss.(37) In animal models, *n*-3 PUFA supplementation also has had a beneficial effect on calcium absorption and balance.(5)

In experimental studies, increasing intake of EPA + DHA has been shown to attenuate bone loss in ovariectomized animal models.(5) The results from the few small intervention trials in postmenopausal women have been mixed, and interpretation of the results is complicated because the supplement protocols often contained other fatty acids, such as the *n*-6 PUFA γ-linolenic acid, in conjunction with fish oil.(7–9) Among observational studies, a high intake of seafood or total *n*-3 PUFAs was associated with greater BMD in elderly men and women in three cross-sectional studies,(10–12) whereas another cross-sectional study found no association between fish intake and BMD in elderly women.(13) A limitation of the cross-sectional studies is that they cannot establish temporality. In a small prospective study among young men, serum long-chain *n*-3 PUFA concentration, and especially DHA concentration, was positively associated with peak BMD and bone accrual.(16) In a case-control study among elderly Japanese men and women, fish consumption 3 to 4 times/week compared with fewer than 2 times/week was associated with 42% (95% CI 9%–64%) lower odds ratio (OR) for hip fracture, but no statistically significant association was found with intake of greater than 4 times/week (OR = 0.70, 95% CI 0.41–1.21).(15) In the Nurses' Health Study, consumption of dark-meat (oily) fish more than 1 serving/week compared with less than 1 serving/month was associated with a relative risk (RR) for hip fracture of 0.67 (95% CI 0.35–1.28, *p* = .03 for trend across the four fish intake groups) among 72,337 postmenopausal women during 18 years of follow-up.(17) In contrast, in the EPIC-Oxford Study of 7947 men and 26,749 women aged 20 to 89 years, consumption of any fish was not associated with the risk of any fractures during the follow-up of 5.2 years.(18) The multivariable-adjusted incident rate ratio in those eating any fish was 1.01 (95% CI 0.88–1.17) compared with the meat eaters. Fish consumption was not associated with hip fracture risk in a cohort of 4573 Japanese elderly men and women either.(19) In summary, the results from the earlier studies have been inconsistent, and the use of different study designs, sites of the BMD measurement, and methods to assess fish intake makes it difficult to compare results. Four studies have investigated the effect of fish consumption on fracture risk, but none has included EPA + DHA intake. Our study is the first that includes information about both fish and EPA + DHA intakes and both BMD and hip fracture incidence in a prospective study with a community-based population of older adults, a population most susceptible to osteoporotic fractures.

It could be speculated that one explanation for the lack of beneficial effect could be too low an intake of fish in this study population. However, the study findings have not been consistent in Japan either, with traditionally high intakes of fish.(11,13,15,19) Another reason why we did not find a beneficial effect with fish or EPA + DHA consumption, not even after stratifying by age, may be the old age (≥65 years at baseline) of the participants. Given the multiple determinants of hip fracture in older people,(38) factors other than fish or PUFA intake may be more important for bone health in the elderly. In experimental studies, environmental pollutants, such as persistent organochlorine compounds (POCs), have been shown to impair bone metabolism,(39,40) but no consistent associations have been seen between fish consumption or POC levels and BMD or risk of osteoporotic fractures in Sweden, where POC contamination of Baltic Sea fish is relatively high.(41,42) Thus, although we cannot exclude competing effects of benefits of EPA + DHA versus POCs as a reason for our null results, such an explanation should be considered speculative, especially because we did not find evidence of dose response with increasing fish or EPA + DHA intake.

Our finding that the estimated EPA + DHA intake was associated with slightly lower BMD in those with a higher LA intake is inconsistent with results from the Rancho Bernardo Study, where a dietary total *n*-6–total *n*-3 PUFA ratio was inversely associated with BMD in older men and women.(14) However, independent associations between either *n*-3 or *n*-6 intake alone and BMD were not reported in that study, and the authors estimated total *n*-3 (EPA + DHA + α-linolenic acid) and total *n*-6 (LA + arachidonic acid) PUFA intakes rather than EPA + DHA and LA, as we did. Using ratios as the exposure makes it difficult to interpret whether the observed associations are due to differences in *n*-3 PUFA intake or *n*-6 PUFA intake or both. The observed interaction between EPA + DHA and LA in our study may only be a chance finding because we did not find evidence for interaction with fish intake or with the risk of hip fracture. Because several associations were evaluated, it is possible that the statistically significant findings may be due to type I error.

The strength of the CHS is the use of a longitudinal design, the population-based recruitment, a large number of participants, and extensive standardized examinations of other risk factors. Potential limitations are also present. Dietary intakes were assessed by a single FFQ at baseline, a method that is imperfect and would result in some exposure misclassification and thus diminish the ability to detect relationships between dietary factors and disease risk. The FFQ was not administered simultaneously with the DXA scan; however, FFQs assess long-term dietary intake habits. We did not have information about the intake of another *n*-6 PUFA, arachidonic acid. However, LA is the major *n*-6 PUFA in the diet, accounting for 85% to 90% of dietary *n*-6 PUFAs.(2) Although the CHS investigators conducted active and passive surveillance to capture all hospitalizations for hip fractures, adjudicated reviews of the diagnoses were not performed. However, misclassification is not likely to be extensive.(43,44)

In conclusion, the results from this study do not support a strong effect of either fish or EPA + DHA consumption on bone health in older men and women, a population most susceptible to osteoporotic fractures. Results from the earlier studies about fish consumption and bone health also have been inconsistent. Given the multiple determinants of hip fractures in older people,(38) these results suggest that other factors than fish consumption may be more important for bone health in this age group.
